# Evaluating the Influence of ipRGCs on Color Discrimination

**DOI:** 10.3390/jimaging8060154

**Published:** 2022-05-28

**Authors:** Masaya Ohtsu, Akihiro Kurata, Keita Hirai, Midori Tanaka, Takahiko Horiuchi

**Affiliations:** 1 Graduate School of Science and Engineering, Chiba University, Yayoi-cho 1-33, Inage-ku, Chiba 263-8522, Japan; kurata89@chiba-u.jp (A.K.); hirai@faculty.chiba-u.jp (K.H.); horiuchi@faculty.chiba-u.jp (T.H.); 2 Graduate School of Global and Transdisciplinary Studies, Chiba University, Yayoi-cho 1-33, Inage-ku, Chiba 263-8522, Japan; midori@chiba-u.jp

**Keywords:** ipRGC, melanopsin, color discrimination, metamer

## Abstract

To investigate the influence of intrinsically photosensitive retinal ganglion cells (ipRGCs) on color discrimination, it is necessary to create two metameric light stimuli (metameric ipRGC stimuli) with the same amount of cone and rod stimulation, but different amounts of ipRGC stimulation. However, since the spectral sensitivity functions of cones and rods overlap with those of ipRGCs in a wavelength band, it has been difficult to independently control the amount of stimulation of ipRGCs only. In this study, we first propose a method for calculating metameric ipRGC stimulation based on the orthogonal basis functions of human photoreceptor cells. Then, we clarify the controllable range of metameric ipRGC stimulation within a color gamut. Finally, to investigate the color discrimination by metameric ipRGC stimuli, we conduct subjective evaluation experiments on 24 chromaticity coordinates using a multispectral projector. The results reveal a correlation between differences in the amount of ipRGC stimulation and differences in color appearance, indicating that ipRGCs may influence color discrimination.

## 1. Introduction

The study of the mechanisms of human color perception and color vision is one of the leading research fields in color science. Several previous studies have suggested that the spectral sensitivity of L, M, and S cones and rods is optimized for natural environments and natural scene statistics [1,2,3,4,5]. We perceive color by the amount of response of the cones and rods, and we can now calculate perceived color on the basis of this response and color appearance models such as CIECAM [6,7]. Color resolving power has also been studied and applied to model development [8,9]. Color discrimination research, which investigates the relationship between quantitative and perceptual differences between colors, is useful in industrial fields that require a high-level assessment of color differences [10,11,12,13]. In the 21st century, an intrinsically photosensitive retinal ganglion cell (ipRGC) was discovered on the retina as the third photoreceptor cell, after cone and rod cells [14]. The ipRGCs have the photoreceptor melanopsin and respond to light stimuli on their own. The ipRGCs are also influenced by cone and rod inputs. Response time is slow, and output is continuous. Further, the ipRGCs influence circadian rhythms and the generation of the pupillary reflex to light [14,15].

The ipRGCs affect not only biological responses but also visual perception. Brown et al. [16] studied the perception of brightness related to the ipRGCs. They used a multi-primary stimulus presentation device with four-color LEDs as the light source, and conducted experiments with stimuli that varied the amount of stimulation to the ipRGCs without changing the amount of stimulation to the cones. As a result, it was confirmed that brightness perception decreased as the amount of stimulation to the ipRGCs increased. Yamakawa et al. [17] formulated brightness perception related to the ipRGCs, by conducting experiments using a stimulus presentation device with six primary colors. These results suggest that even metameric stimuli (despite having different spectral distributions) perceive brightness differently. Recently, the influence of the ipRGCs on color perception has also been investigated. Cao et al. [18] studied the contribution of the ipRGCs to color perception in response to a white light source by independently controlling the stimulation of each photoreceptor cell using five-primary color light source devices. The results suggest that the ipRGC response affects the yellowness of white light.

Most of the previous studies on the visual function of ipRGCs have been conducted using multi-primary color light source devices. The multi-primary color method can be used to conduct experiments with stable and high-brightness light sources, so it is a major light source method in studies of color vision. However, the drawback of this method is that the gamut range that can be created is limited by the primary source. Moreover, the controllable range of the amount of stimulus to the ipRGCs is limited by the primary source; therefore, it is difficult to create stimuli that maximally change the ipRGC stimulation amount at a certain chromaticity.

This paper presents a study of the influence of the ipRGCs on color discrimination using a spectral projector [19] constructed in the author’s laboratory. The spectral projector can output light sources with arbitrary spectral characteristics, allowing more detailed experiments than multi-primary color light source devices. Although there are several possible situations of color discrimination, this study deals with color discrimination presented in a temporal sequence. Specifically, we first propose a simple and accurate method for independently controlling the amount of stimulus given to the ipRGCs using the spectral sensitivity functions of the cones, rods, and ipRGCs. In addition, we clarify the chromaticity range over which the ipRGC stimulation amount can be independently controlled. Finally, we conduct color discrimination experiments using pairs of stimuli in which only the ipRGC stimulation amount is varied (metameric ipRGC stimuli), and then discuss the influence of the ipRGCs on color discrimination by examining the relationship between the difference in the ipRGC stimulation amount for each stimulus and the participants’ discrimination rate.

## 2. Metameric ipRGC Stimuli

We derived metameric ipRGC stimuli used in this study. Metameric ipRGC stimuli are a pair of stimuli using the same amounts of cone and rod stimulation, but different amounts of ipRGC stimulation. First, we describe the method to control independent ipRGC stimulation in Section 2.1. Then, we describe the range of independent control of the amount of ipRGC stimulation for each chromaticity coordinate in Section 2.2.

### 2.1. Independent Control of ipRGC Stimuli Using Spectral Basis

**Figure 1:**
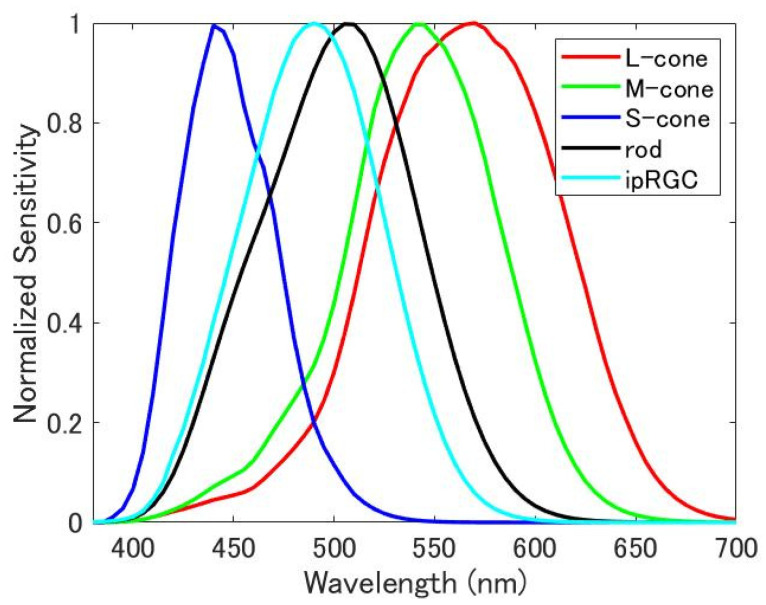
Spectral sensitivity function of the cone, rod, and ipRGC.

**Figure 2:**
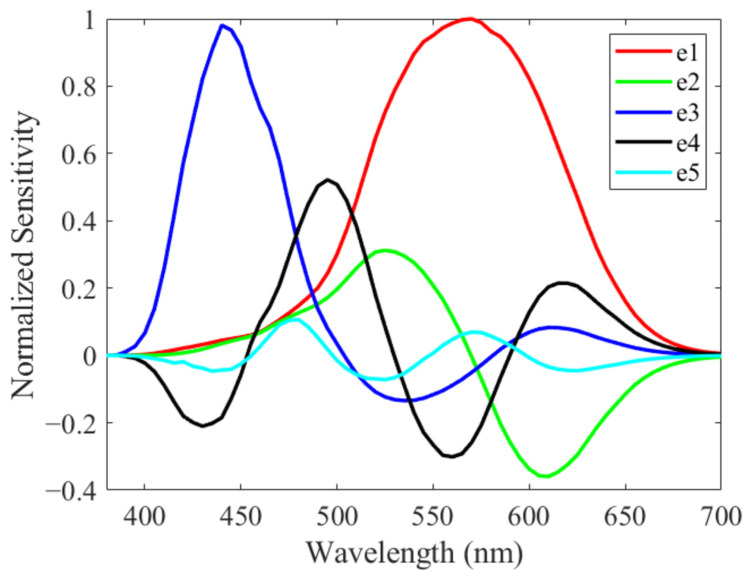
Orthonormal basis $e_{1}$ to $e_{5}$.

Figure 1 shows the spectral sensitivity functions of LMS cones, rods, and ipRGCs. Here, spectral sensitivities of LMS cones (l(λ), m(λ), and s(λ)) are derived from CIE170-1:2006 LMS (for a standard observer 22 years old, with a 2-degree field of view [20]), and the sensitivity of ipRGC is defined by the Commission Internationale de l’Eclairage (CIE) [21]. From these functions, the bases ($e_{1}$ to $e_{5}$) were obtained by orthonormalizing the L-cone, M-cone, S-cone, rod, and ipRGC, in this order (Figure 2). The spectral distribution p(λ) of the light stimulus was created by a weighted linear sum using each basis $e_{1}$ to $e_{5}$ and coefficients $\omega_{1}$ to $\omega_{5}$ as follows:

(1) $$p\left(\lambda \right){=\omega }_{1}e_{1}\left(\lambda \right){+\omega }_{2}e_{2}\left(\lambda \right){+\omega }_{3}e_{3}\left(\lambda \right){+\omega }_{4}e_{4}\left(\lambda \right){+\omega }_{5}e_{5}\left(\lambda \right).$$

Here, the L-cone, M-cone, S-cone, rod, and ipRGC are orthonormalized in this order to obtain the basis. Therefore, the spectral sensitivity functions of the L-cone, M-cone, S-cone, rod, and ipRGC were calculated by using the basis and the coefficient matrix as follows:

(2) $$\left[l\left(\lambda \right)\;m\left(\lambda \right)\;s\left(\lambda \right)\;r\left(\lambda \right)\;i\left(\lambda \right)\right]=\left[e_{1}\left(\lambda \right){\;e}_{2}\left(\lambda \right){\;e}_{3}\left(\lambda \right){\;e}_{4}\left(\lambda \right){\;e}_{5}\left(\lambda \right)\right]\;\alpha ,\;\alpha =(\begin{array}{ccccc} 1 & 0.8046 & 0.0518 & 0.4946 & 0.3296\\ 0 & 1 & 0.3310 & 1.5771 & 1.3412\\ 0 & 0 & 1 & 0.4435 & 0.6118\\ 0 & 0 & 0 & 1 & 1.1761\\ 0 & 0 & 0 & 0 & 1 \end{array})$$

When the light stimulus p(λ) is incident on the retina, the respective stimulus amounts of the L-cone, M-cone, S-cone, rod, and ipRGC (L, M, S, R, I) can be calculated using the spectral sensitivity functions and the spectral distributions of the light stimulus.

Since the bases $e_{1}$ to $e_{5}$ are orthonormal bases, using Equations (1) and (2), L, M, S, R, and I can be expressed as follows:

(3) $$\begin{array}{cc} \left[L\;M\;S\;R\;I\right] & =\left[l\left(\lambda \right)\;m\left(\lambda \right)\;s\left(\lambda \right)\;r\left(\lambda \right)\;i\left(\lambda \right)\right]\;p\left(\lambda \right)\\ & =\left[e_{1}\left(\lambda \right){\;e}_{2}\left(\lambda \right){\;e}_{3}\left(\lambda \right){\;e}_{4}\left(\lambda \right){\;e}_{5}\left(\lambda \right)\right]\;\alpha \;\left\{\omega_{1}e_{1}\left(\lambda \right){+\omega }_{2}e_{2}\left(\lambda \right){+\omega }_{3}e_{3}\left(\lambda \right){+\omega }_{4}e_{4}\left(\lambda \right){+\omega }_{5}e_{5}\left(\lambda \right)\right\}\\ & =\left[\omega_{1}{\;\omega }_{2}{\;\omega }_{3}{\;\omega }_{4}{\;\omega }_{5}\right]\;\alpha . \end{array}$$

Therefore, the (relative) stimulus amounts L, M, S, R, and I of each photoreceptor cell can be expressed by the following equations.

(4) $$\begin{array}{cc} {L=\omega }_{1}, & \\ M={0.8046\;\times \;\omega }_{1}{+\omega }_{2}, & \\ S={0.0518\;\times \;\omega }_{1}+{0.3310\;\times \;\omega }_{2}{+\omega }_{3}, & \\ R={0.4946\;\times \;\omega }_{1}+{1.5771\;\times \;\omega }_{2}+{0.4435\;\times \;\omega }_{3}{+\omega }_{4}, & \\ I={0.3296\;\times \;\omega }_{1}+{1.3412\;\times \;\omega }_{2}+{0.6118\;\times \;\omega }_{3}+{1.1761\;\times \;\omega }_{4}{+\omega }_{5}. & \end{array}$$

As shown in Equation (4), the amount of ipRGC stimulation depends on $\omega_{5}$, which is the coefficient of the base $e_{5}$. Therefore, the spectral stimulus generated by changing only $\omega_{5}$ becomes an ipRGC metameric stimulus. Then, it becomes possible to control the amount of ipRGC stimulation independently.

### 2.2. Color Gamut Simulation Due to Metameric ipRGC Stimuli

**Figure 3:**
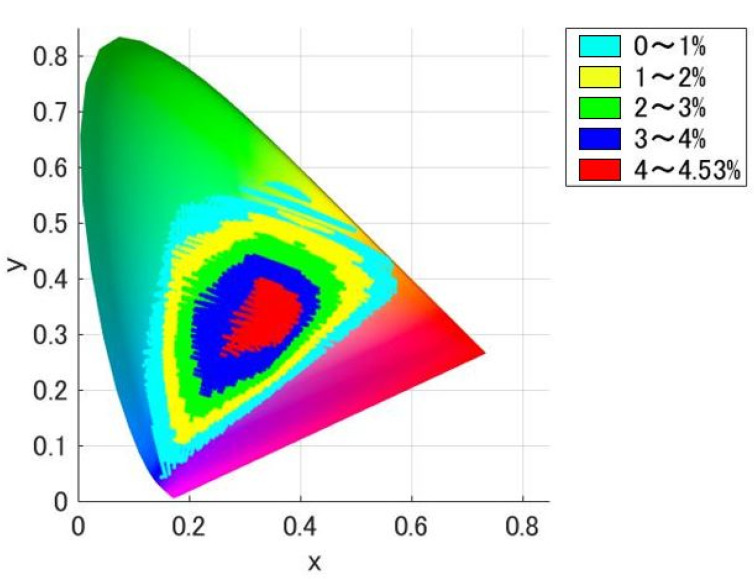
The chromaticity of metameric ipRGC stimuli. Only the ipRGCs can become variables in the region. In the order of red, blue, green, yellow, and light blue, metameric amounts of ipRGC stimulation become larger.

As shown in the orthonormal basis functions of Figure 2, since $e_{1}$ to $e_{5}$ contains negative values, a light stimulus p(λ) often becomes negative in value. A negative optical stimulus (spectral distribution) cannot be projected in the experiment. Therefore, it is necessary to find the presentable metameric ipRGC stimulus p(λ), the minimum value of which is 0 or more in the wavelength region. Figure 3 shows all non-negative ipRGC metameric stimuli (spectral distribution) plotted on an xy chromaticity diagram using CIE2015XYZ [20]. The color-coding indicates how much the ipRGC alone can be changed. Since there is no general method for quantifying the ipRGC response, in the study, we define the ipRGC stimulation amount I (%) of the spectral distribution p(λ) as follows:

(5) $$I=100\frac{\int_{\lambda }^{}p\left(\lambda \right)i\left(\lambda \right)d\lambda }{\int_{\lambda }^{}p_{w}\left(\lambda \right)i\left(\lambda \right)d\lambda },$$

where i(λ) is the spectral sensitivity function of ipRGC, and $p_{w}\left(\lambda \right)$ is the spectral distribution of white light, which is 100 in the wavelength range. On the other hand, the ipRGC stimulation amount is defined as 100 for the white light (the spectral distribution of which is 100 in the wavelength range). The color code indicates the percentage of changes that can be made only to ipRGC (light blue at 0% to 1% chromaticity, yellow at 1% to 2% chromaticity, green at 2% to 3% chromaticity, blue at 3% to 4% chromaticity, and red at 4% to 4.53% (maximum) chromaticity).

As shown in Figure 3, it can be confirmed that the closer the light stimulus is to the white point, the more the amount of ipRGC stimulus can be controlled independently (in other words, the easier it is to create the ipRGC metameric stimulus). Additionally, the independent control of ipRGC is possible with more chromaticity in blue stimuli than in green and red stimuli.

Note that the range over which only ipRGC can be modulated, shown in Figure 3, does not consider all stimuli. It is possible to generate ipRGC components with a numerically wider amplitude by not limiting the original 5 dim space of the cones, rods and ipRGC. However, the performance of our system is limited by the inability to represent spectral distributions with steep changes. Therefore, even if the numerical values were good, we had to select the stimuli to be used, so we derived it from the original 5 dim space.

## 3. Experimental Methods

This chapter describes the methods of visual evaluation experiments conducted to investigate the influence of ipRGCs on color discrimination. First, we describe the chromaticity coordinates of the metameric ipRGC stimuli used in the experiment in Section 3.1. Then, we describe the details of the experimental procedure in Section 3.2.

### 3.1. Experimental Stimuli

For the experimental stimuli, the following six groups were selected.

Group 1. red to purple hue
Group 2. green hue
Group 3. orange hue
Group 4. blue to purple hue
Group 5. blue hue
Group 6. yellow-green hue

**Figure 4:**
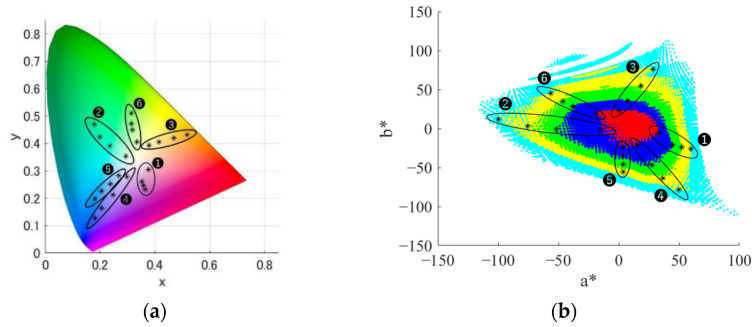
Actual experimental stimuli of 24 chromaticity points. (**a**) xy chromaticity diagram. (**b**) a*b* chromaticity diagram.

**Figure 5:**
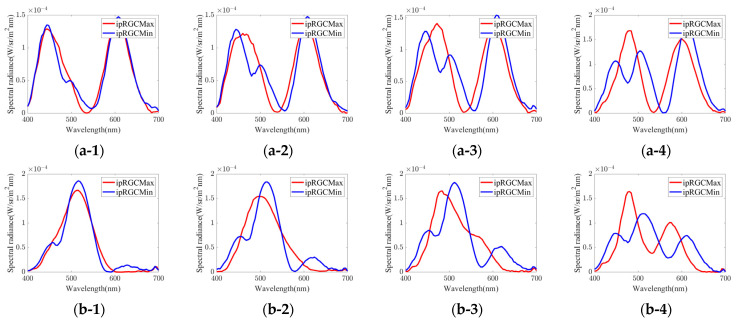

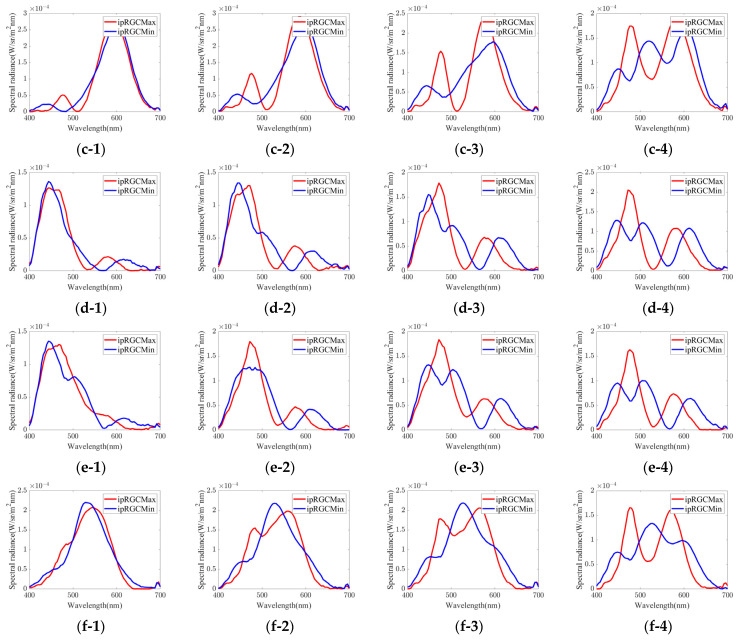
Spectral distribution of actual metameric ipRGC stimuli in the experiments (measured by a spectroradiometer); (**a1**–**a4**) Group 1; (**b1**–**b4**) Group 2; (**c1**–**c4**) Group 3; (**d1**–**d4**) Group 4; (**e1**–**e4**) Group 5; (**f1**–**f4**) Group 6. In addition, the numbers indicate the difference in the ipRGC stimulation amount for each theoretical stimulus; (x**-1**) ipRGC diff. = 1%; (x**-2**) ipRGC diff. = 2%; (x**-3**) ipRGC diff. = 3%; (x**-4**) ipRGC diff. = 4% (x means caption alphabetical (a, b, …, f)).

These groups were within the range where independent control of the ipRGC stimulation amount is possible by 1% or more. For each group, we selected 4 points from white point to highly saturated chromaticity. Along the independently controllable range of the ipRGC stimulation amount for each chromaticity shown in Figure 3, the points were selected so that the controllable range of the ipRGC stimulation amount for each chromaticity was approximately 1, 2, 3, and 4%. Figure 4 shows the 24 color chromaticity points selected for the actual experimental stimuli plotted on xy and a*b* chromaticity diagrams. We tried to set them as evenly as possible in the equal color space. However, as shown in Figure 4b, the regions were unequally distributed, and it was theoretically impossible to set all of them at equal intervals. Therefore, we set them approximately evenly. All stimuli were calibrated before the experiment and adjusted so that ${\Delta L}_{\mathrm{ab}}\;<\;2$. In addition, after the experiment, we measured again to confirm that there were no significant changes in the stimuli during the experiment. Figure 5, Table 1 and Table 2 show the metameric ipRGC stimuli in the experiment. We measured the actual spectral distributions and CIELMS (CIEXYZ), rod, and ipRGC stimulation amounts with a spectroradiometer (PR-655, Photo Research, Inc., Chatsworth, California, USA). Measurements were taken twice per session, and the average value confirmed that the condition ${\Delta L}_{\mathrm{ab}}\;<\;2\;$was met. Figure 5 shows the spectral distribution of actual metameric ipRGC stimuli in the experiments. Table 1 shows Y (Luminance), x, y, L-cone, M-cone, S-cone, rod, and ipRGC stimulation amounts for actual stimuli, and Table 2 shows the theoretical values. As well as the ipRGC stimulation amount, the cone and rod stimulation amounts were defined as 100 for the reference white light, which was 100 in the wavelength range. As shown in Table 1 and Table 2, there were differences between the theoretical and measured xy chromaticity coordinates for some of the stimuli. These were the results of careful experimentation with our system and were the limitations of precision adjustment. On the other hand, our system was able to generate light sources with sufficiently good accuracy compared to previous studies using multi-primary methods such as LED lights. Furthermore, since the difference in the amount of ipRGC stimulation was larger than the difference in the amount of cone and rod stimulation, we judged that these experimental stimuli were consistent with the experimental design. Additionally, the differences in the rod and ipRGC stimulation amounts were due to the difference in the reference white light source.

### 3.2. Procedure

In this study, we used a multispectral projector [19] as the experimental stimuli presentation device. The light source component of the projector consisted of an OL490 (Optronic Laboratories, Orlando, FL, USA), which was programmable using a computer. The system used a chip with 1024 × 768 pixels, where the former number influences the wavelength resolution in the range of 380–780 nm and the latter number determines the intensity quantization level. In this article, the sampling pitch for calculating the spectra was set at an interval of 5 nm. The image projection component of our prototype was based on a Texas Instruments DLP Lightcrafter (Dallas, TX, USA). The original LED-based RGB primary colors were replaced with a spectral light source. The spectral light source was illuminated on a DMD chip for image projection through a condenser lens and fly array lens. The micromirrors on the DMD chip also had two on–off bi-stable states that, in this case, controlled the spatial image projections with an 8-bit depth. The present system used a chip with 608 × 684 pixels for image projection. In this study, each experimental stimulus was created with a wavelength interval of 4 nm, and each wavelength was controlled in the range of 0–100% output power.

**Figure 6:**
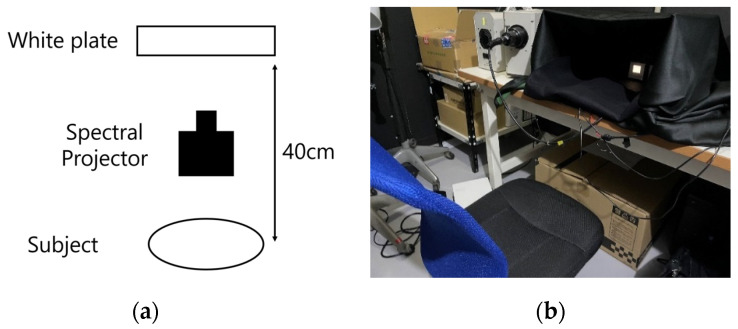
Experiment environment. (**a**) Bird’s-eye view. (**b**) Experiment environment.

Figure 6 shows the experimental environment. We experimented in a dark room, creating experimental stimuli with a multispectral projector and projecting the same on a white plate made by Konica Minolta. The distance from the stimulus presentation position (white version) to a participant was 40 cm. The stimulus was a square, measuring 3 cm long and 3 cm wide. Thus, the experimental viewing angle was 4.3 degrees. The participant looked at the center of the stimulus.

**Figure 7:**
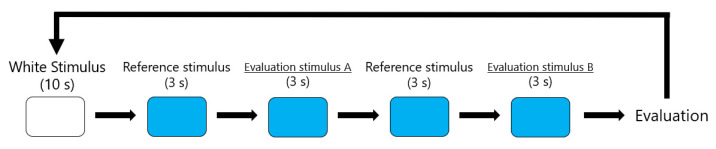
Stimulus presentation procedure.

We conducted experiments with each of the 24 points on the xy chromaticity diagram. The participants were five men in their twenties, and were confirmed by the Ishihara test to have normal color vision. To prevent the effects of ipRGCs on circadian rhythms from contributing to the experimental results, all experiments were conducted during the daytime. There were two types of evaluation stimuli: the maximum metameric ipRGC stimulus that maximizes the amount of ipRGC stimulation at each chromaticity point, and the dummy stimulus that was the same as the reference. The stimulus presentation procedure is shown in Figure 7. Participants first observed a white light with a spectral component of all 100 for 10 s. After the reference stimulus (the minimum metameric ipRGC stimulus) was presented for 3 s, ipRGC metameric stimuli or dummy stimuli were presented randomly for 3 s. The participants responded on the basis of the difference they felt in color appearance compared to the reference stimulus. The number of trials was two for each stimulus pair.

## 4. Results

**Figure 8:**
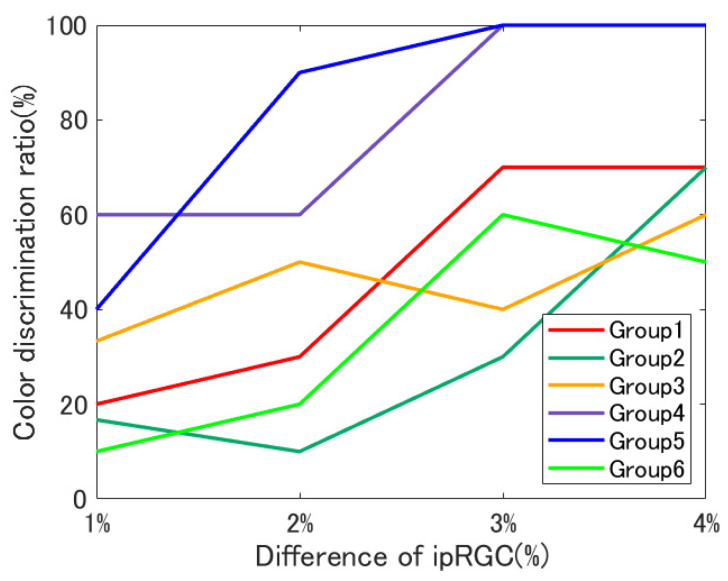
Experimental results.

Figure 8 shows the color discrimination experiment results. The horizontal axis shows the difference in the amount of ipRGC stimulation between the metameric ipRGC stimuli, and the vertical axis shows the percentage of participants perceiving a difference in color vision.

**Figure 9:**
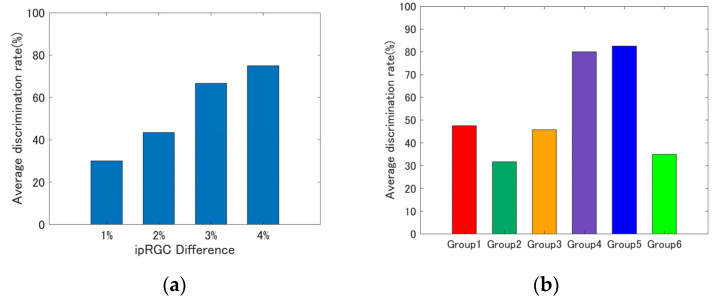
Average of experimental results; (**a**) each difference in the amount of ipRGC stimulation; (**b**) each group.

Figure 9 shows the average of the experimental results. Figure 9a shows the averages for each difference in the amount of ipRGC stimulation (theoretical value), and Figure 9b shows the averages for each group. If the color matching function included a contribution from the ipRGCs, then color appearance should match for all experimental stimuli, regardless of the difference in the ipRGC stimulation amounts. However, as shown in Figure 9a, the larger the difference in the amount of ipRGC stimulation, the more the participants perceived a difference in color vision. Additionally, as shown in Figure 9b, participants perceived differences in color vision for the blue hue compared to the red and green hues. However, several concerns exist in this study, and these are discussed below.

### 4.1. Individual Differences

The metameric ipRGC stimuli in the previous section were based on the LMS cone spectral sensitivity function of a standard observer (CIE2006LMS, 22 years old, a 2-degree field of view). However, it is known that individual differences exist in cone spectral sensitivity. Even if the stimuli are metameric for a standard observer, the participants’ individual differences in cone spectral sensitivity may prevent metamerism from being established, and this lack of metamerism may affect discrimination. Therefore, an additional experiment was conducted on the participants to investigate the effect of individual differences on the experimental results.

As an experimental method, we created stimulus pairs in which the cone, rod, and ipRGC stimulus amounts were all the same (pentamic-metamer stimuli [11]), and we then investigated whether participants could color-discriminate the stimulus pairs. If a participant could color-discriminate pentameric metameric stimuli, then the participant was capable of color discrimination, even though the colors were the same for standard observers, and individual differences in visual characteristics may affect color discrimination. The results of the experiment of metameric ipRGC stimuli confirmed that two of the five participants might have been affected by individual differences in visual characteristics. In addition, in the remaining three participants, it was confirmed that the results of the Group 4 experiment might have been affected by individual differences in visual characteristics. See Appendix A for details of the experiment.

### 4.2. Luminance of Experimental Stimuli

In this experiment, in order to output the created metameric ipRGC stimuli with higher precision, we used a spectral projector as the light source presentation device. However, as can be seen in Table 1, the luminance Y of the experimental stimuli was very low, ranging from about 1 to 10 cd/m^2^. Therefore, it was difficult to quantify the adaptation status of the eye, and this made it necessary to match the amount of rod stimulation when creating metameric ipRGC stimuli, thus reducing the control range of the ipRGC stimulation amount. When rod stimulation amount matching was considered, the maximum controllable range of the ipRGC stimulation amount was 4.53%; if it was not considered, the maximum was 30.7%. In the future, it will be possible to investigate this question more clearly using a light source with higher luminance and precision.

### 4.3. Viewing Angle

Finally, the viewing angle of the experimental stimuli must also be considered. In this study, stimuli were created using 2-degree spectral sensitivity function, but due to the experimental environment, they were presented with a 4.3-degree field of view. Therefore, we calculated the color difference ΔL_ab_ for each experimental stimulus using a 4.3-degree color matching function derived from CIE 170-1:2006 [20] and investigated its effect on the experiment. Table 3 shows the color differences for the metameric ipRGC stimuli (Section 3), and Table 4 shows the color differences for the pentameric metameric stimuli (Section 4.1). As shown in Table 3 and Table 4, all but one of the stimuli achieved ${\Delta L}_{\mathrm{ab}}\;<\;2$, which we set as the criterion for calibration accuracy. Therefore, we confirmed that this did not affect our previous manuscript’s assertions.

In addition, previous studies have confirmed that the ipRGCs are distributed mainly in the peripheral visual field. Therefore, in the future, it will be necessary to create experimental stimuli with cone spectral sensitivity functions for a 10-degree field of view and conduct experiments in a peripheral field of view environment.

## 5. Conclusions

**Figure 10:**
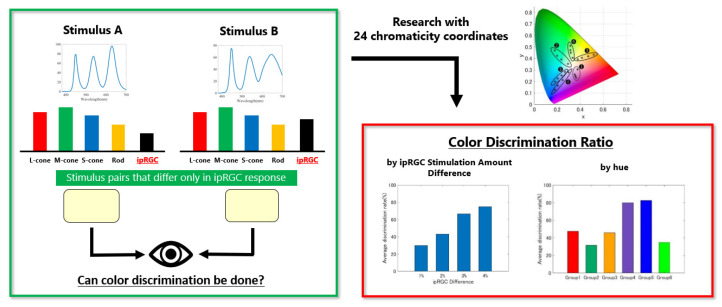
Graphic summary of this paper.

The purpose of this study was to clarify the effect of metameric ipRGC stimuli on the chromaticity range and color discrimination. In order to achieve this goal, first, we pro-posed a method to control the amount of ipRGC stimulation independently and then showed the color gamut due to metameric ipRGC stimuli. Second, we conducted color discrimination experiments. The results suggested that the closer the hue was to blue, the easier it was to present the metameric ipRGC stimulus, and the higher the effect of the ipRGC on color discrimination. Furthermore, it was suggested that the larger the difference in the amount of ipRGC stimulation, the higher the effect of the ipRGC on color perception. Finally, we examined individual differences in color vision among the participants to confirm that these results could not be explained solely by individual differences. Figure 10 shows the graphic summary of this paper.

The improved results are summarized as follows:

- Previous studies on the contribution of ipRGCs to color perception have all, as far as the author has been able to determine, been conducted using white stimuli. This study is the first to investigate the contribution of ipRGCs to color vision for a number of color stimuli other than white and the first to experimentally demonstrate that ipRGCs may influence color discrimination, particularly for blue stimuli.
- The multispectral projector used in this experiment could present color stimuli with far greater precision than previous multi-primary methods. To investigate the contribution of ipRGCs to color perception using this device, it was necessary to be able to independently control the ipRGC stimulation amount in the spectral data. The method proposed in Section 2 solved this problem. We expect that it will be possible to investigate the contribution of ipRGCs to vision with higher precision than in previous studies, not only in this study’s experiments.

In future research, we intend to conduct a similar experiment with a larger number of participants and with peripheral vision.

## Figures and Tables

**Table 1:** Y (luminance), x, y, ${\Delta L}_{\mathrm{ab}}$, cone, rod, and ipRGC stimulation amount of actual experimental stimuli (measured by a spectroradiometer). The rod and ipRGC stimulation amounts are defined as 100 for a light source presented by a spectral projector with white light the spectral distribution, which is 100 in the wavelength range.

Group	Chromaticity Point	ipRGC diff. = 1%		ipRGC diff. = 2%		ipRGC diff. = 3%		ipRGC diff. = 4%	
	ipRGC	Max	Min	Max	Min	Max	Min	Max	Min
**Group 1**	**Y (cd/m^2^)**	4.10	4.12	4.22	4.27	4.60	4.68	5.42	5.46
	**x**	0.364	0.365	0.356	0.358	0.353	0.353	0.374	0.376
	**y**	0.233	0.235	0.246	0.247	0.260	0.259	0.304	0.303
	**ΔL_ab_**	1.17		0.724		0.598		1.45	
	**L-cone (%)**	21.8	21.7	22.2	22.0	24.1	23.7	27.9	27.6
	**M-cone (%)**	17.3	17.2	19.0	18.6	21.5	21.1	25.5	25.5
	**S-cone (%)**	60.9	59.6	59.2	57.7	59.5	58.9	49.6	49.0
	**rod (%)**	22.2	21.7	26.3	25.5	29.8	29.3	33.4	33.8
	**ipRGC (%)**	27.9	26.5	32.8	30.4	36.8	34.4	40.4	37.9
**Group 2**	**Y (cd/m^2^)**	5.03	5.03	5.00	5.00	5.35	5.28	4.80	4.72
	**x**	0.179	0.180	0.202	0.200	0.231	0.235	0.290	0.294
	**y**	0.471	0.475	0.425	0.424	0.393	0.392	0.353	0.357
	**ΔL_ab_**	0.870		0.536		1.77		1.33	
	**L-cone (%)**	21.7	21.7	21.9	21.8	23.6	24.0	22.2	22.5
	**M-cone (%)**	33.9	33.9	32.8	32.9	33.3	33.6	27.0	27.5
	**S-cone (%)**	31.9	31.3	37.6	37.8	43.3	43.5	39.5	41.3
	**rod (%)**	45.8	46.1	46.3	46.3	46.5	47.2	35.3	34.7
	**ipRGC (%)**	47.3	46.8	49.8	48.1	51.1	49.6	40.2	36.8
**Group 3**	**Y (cd/m^2^)**	9.93	9.99	11.1	11.0	8.74	8.63	8.79	8.83
	**x**	0.518	0.518	0.467	0.470	0.415	0.416	0.376	0.378
	**y**	0.432	0.435	0.420	0.421	0.406	0.408	0.393	0.397
	**ΔL_ab_**	1.73		1.43		0.974		1.54	
	**L-cone (%)**	52.5	52.2	56.8	56.9	43.3	43.8	43.4	43.3
	**M-cone (%)**	43.0	42.5	50.4	50.8	42.5	43.0	45.6	45.4
	**S-cone (%)**	9.35	9.78	24.4	25.5	31.8	33.0	42.8	44.1
	**rod (%)**	18.7	18.9	29.5	29.0	30.9	30.9	44.3	44.3
	**ipRGC (%)**	13.8	12.5	26.3	23.3	30.7	27.5	46.7	43.5
**Group 4**	**Y (cd/m^2^)**	1.54	1.51	2.04	2.07	3.15	3.24	4.57	4.71
	**x**	0.182	0.181	0.206	0.205	0.242	0.248	0.296	0.298
	**y**	0.127	0.125	0.165	0.165	0.212	0.214	0.279	0.281
	**ΔL_ab_**	0.484		0.565		1.89		0.996	
	**L-cone (%)**	5.94	5.79	8.55	8.53	15.3	14.8	22.8	22.1
	**M-cone (%)**	8.79	8.56	11.5	11.5	18.3	18.1	25.3	24.6
	**S-cone (%)**	62.9	62.6	61.2	61.6	69.9	69.6	60.3	59.5
	**rod (%)**	24.4	24.1	26.2	26.9	35.7	36.6	39.9	41.2
	**ipRGC (%)**	32.9	31.8	34.5	34.0	46.0	44.5	49.8	47.2
**Group 5**	**Y (cd/m^2^)**	2.40	2.40	2.75	2.87	3.76	3.74	3.25	3.30
	**x**	0.177	0.181	0.206	0.211	0.233	0.237	0.266	0.268
	**y**	0.198	0.200	0.225	0.227	0.252	0.254	0.282	0.281
	**ΔL_ab_**	1.27		1.55		1.47		1.30	
	**L-cone (%)**	10.4	10.4	12.9	12.3	17.2	17.2	15.5	15.3
	**M-cone (%)**	16.0	15.9	17.9	17.3	22.3	22.6	18.8	18.6
	**S-cone (%)**	64.6	63.4	61.0	59.7	63.9	65.7	45.3	44.4
	**rod (%)**	34.8	33.8	38.2	39.7	42.5	42.3	32.4	32.6
	**ipRGC (%)**	43.2	41.0	48.7	48.7	52.4	49.5	40.2	37.3
**Group 6**	**Y (cd/m^2^)**	9.92	9.99	10.1	10.1	10.3	10.3	6.95	7.13
	**x**	0.313	0.313	0.315	0.314	0.317	0.318	0.331	0.334
	**y**	0.510	0.512	0.473	0.476	0.449	0.454	0.406	0.409
	**ΔL_ab_**	0.560		1.44		1.89		1.59	
	**L-cone (%)**	46.2	45.9	46.9	47.0	48.3	48.4	34.0	33.1
	**M-cone (%)**	59.2	58.8	58.9	59.2	59.6	59.8	39.5	38.6
	**S-cone (%)**	29.5	28.8	38.7	38.1	45.9	44.3	38.4	38.5
	**rod (%)**	60.5	60.1	63.4	64.0	65.8	66.4	43.1	44.0
	**ipRGC (%)**	56.6	54.9	62.4	60.7	66.4	64.1	45.9	43.5

**Table 2:** x, y, cone, rod, and ipRGC stimulation amounts of theoretical and experimental stimuli. The rod and ipRGC stimulation amounts are defined as 100 for the virtual white light, the spectral distribution of which is in the 100 wavelength range.

Group	Chromaticity Point	ipRGC Diff. = 1%		ipRGC Diff. = 2%		ipRGC Diff. = 3%		ipRGC Diff. = 4%	
	ipRGC	Max	Min	Max	Min	Max	Min	Max	Min
**Group 1**	**x**	0.357		0.347		0.343		0.362	
	**y**	0.218		0.227		0.241		0.286	
	**L-cone (%)**	40.5		40.5		44.2		44.2	
	**M-cone (%)**	30.2		32.4		37.1		38.2	
	**S-cone (%)**	70.1		67.9		68.8		49.1	
	**rod (%)**	36.8		42.3		48.4		46.9	
	**ipRGC (%)**	44.1	43.1	50.4	48.4	57.3	54.2	54.2	50.2
**Group 2**	**x**	0.172		0.191		0.221		0.282	
	**y**	0.448		0.400		0.371		0.338	
	**L-cone (%)**	33.2		33.2		36.8		36.8	
	**M-cone (%)**	48.0		46.3		48.3		41.8	
	**S-cone (%)**	28.6		34.5		40.8		40.7	
	**rod (%)**	59.7		60.5		62.7		49.8	
	**ipRGC (%)**	58.9	57.8	62.0	59.9	66.0	62.8	54.3	50.2
**Group 3**	**x**	0.511		0.462		0.406		0.366	
	**y**	0.432		0.420		0.402		0.385	
	**L-cone (%)**	47.9		55.3		51.6		66.3	
	**M-cone (%)**	36.4		45.8		47.1		64.9	
	**S-cone (%)**	5.86		14.6		23.6		40.5	
	**rod (%)**	14.9		24.5		31.7		58.8	
	**ipRGC (%)**	10.7	9.68	20.9	18.8	30.2	27.1	59.3	55.3
**Group 4**	**x**	0.180		0.204		0.236		0.288	
	**y**	0.107		0.145		0.189		0.263	
	**L-cone (%)**	11.1		14.7		22.1		29.5	
	**M-cone (%)**	15.3		18.3		25.1		30.7	
	**S-cone (%)**	71.3		64.6		65.1		48.0	
	**rod (%)**	35.8		36.1		42.6		42.2	
	**ipRGC (%)**	45.5	44.4	45.0	43.0	52.0	49.0	49.6	45.6
**Group 5**	**x**	0.175		0.201		0.227		0.259	
	**y**	0.177		0.203		0.230		0.264	
	**L-cone (%)**	14.7		18.4		25.8		25.8	
	**M-cone (%)**	21.1		24.2		31.4		29.3	
	**S-cone (%)**	55.9		52.6		60.1		45.4	
	**rod (%)**	38.4		44.8		50.2		42.5	
	**ipRGC (%)**	45.0	43.9	53.7	51.7	58.7	55.6	49.8	45.8
**Group 6**	**x**	0.301		0.301		0.304		0.321	
	**y**	0.500		0.465		0.440		0.395	
	**L-cone (%)**	59.0		59.0		62.6		55.3	
	**M-cone (%)**	70.1		69.0		72.1		60.0	
	**S-cone (%)**	22.8		29.0		35.8		39.2	
	**rod (%)**	60.0		62.3		67.0		56.1	
	**ipRGC (%)**	53.0	51.8	57.9	55.7	64.1	61.1	56.6	52.5

**Table 3:** The color difference ${\Delta L}_{\mathrm{ab}}$ (the metameric ipRGC stimuli, calculated using 4.3-degree color matching function).

	Group	ipRGC Diff. = 1%	ipRGC Diff. = 2%	ipRGC Diff. = 3%	ipRGC Diff. = 4%
**ΔL_ab_**	**Group 1**	1.13	0.576	0.758	0.705
	**Group 2**	1.36	1.64	0.306	1.42
	**Group 3**	1.63	0.990	1.53	1.67
	**Group 4**	0.825	1.61	0.740	1.59
	**Group 5**	0.889	0.832	0.975	0.776
	**Group 6**	0.730	1.91	2.43	1.46

**Table 4:** The color difference ΔL_ab_ (the pentamic-metamer stimuli, calculated using 4.3-degree color matching function).

	Group 1	Group 2	Group 3	Group 4	Group 5	Group 6
**ΔL_ab_**	1.37	1.53	1.80	0.406	0.677	1.42

**Figure A1:**
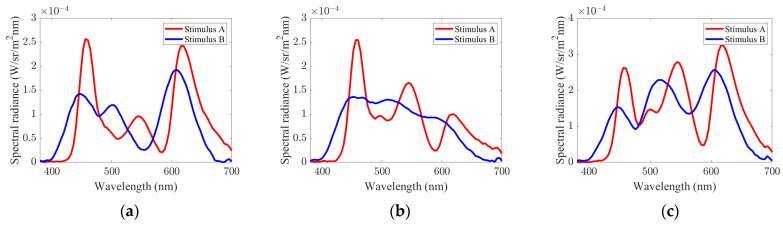

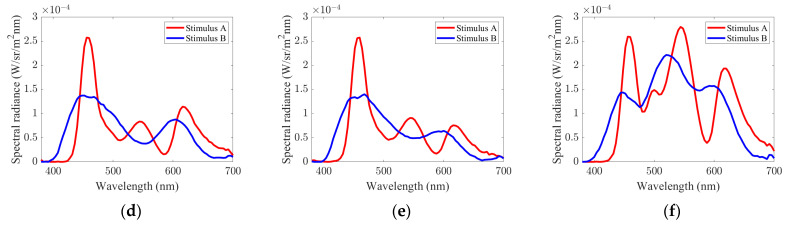
Spectral distribution of actual pentamic-metamer stimuli in the experiments. (**a**) Group 1, ipRGC diff. = 4%; (**b**) Group 2, ipRGC diff. = 4%; (**c**) Group 3, ipRGC diff. = 4%; (**d**) Group 4, ipRGC diff. = 4%; (**e**) Group 5, ipRGC diff. = 4%; (**f**) Group 6, ipRGC diff. = 4%.

**Figure A2:**
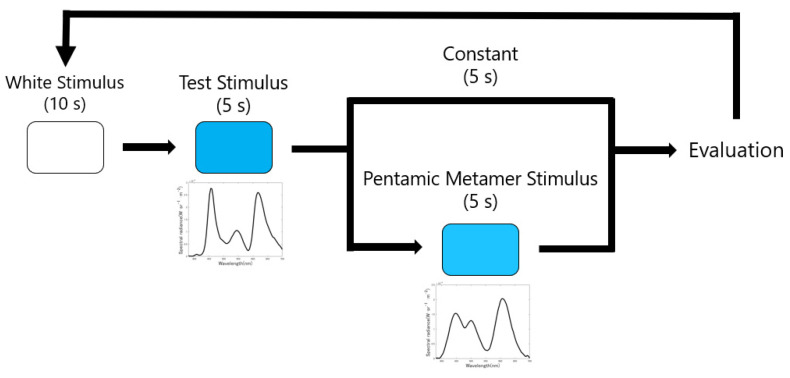
Presentation procedure for pentamic-metamer stimuli experiment.

**Figure A3:**
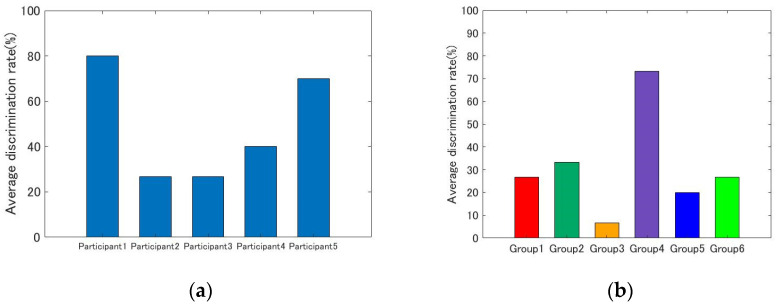
Average of experimental results: (**a**) each participant; (**b**) each Group (only participants 2, 3, and 4).

## Data Availability

Data are contained within the article.

## Appendix A

This appendix presents the color discrimination experiments of pentamic-metamer stimuli, which were conducted to investigate individual differences in visual characteristics among participants. As experimental stimuli, we created stimulus pairs in which the amount of cone, rod, and ipRGC stimulation was the same (pentamic-metamer stimuli [11]) for the chromaticity coordinate of the metameric ipRGC stimuli in which the ipRGC difference was 4% for each group. Figure A1, Table A1 and Table A2 show the pentamic-metamer stimuli in the experiment. Figure A1 shows the spectral distribution of actual pentamic-metamer stimuli in the experiments. Table A1 presents Y (luminance), x, y, L-cone, M-cone, S-cone, rod, and ipRGC stimulation amounts of actual stimuli, and Table A2 shows the theoretical values. For Table A1 and Table A2, the difference between the theoretical and measured values for the rod and ipRGC stimulation amounts is due to the difference in the reference white light source.

**Table A1:** Y (luminance), x, y, ΔL_ab_, cone, rod, and ipRGC stimulation amounts of actual pentamic-metamer stimuli (measured by a spectroradiometer). The rod and ipRGC stimulation amounts are defined as 100 for a light source presented by a spectral projector with white light, the spectral distribution of which is 100 in the wavelength range.

	Group 1		Group 2		Group 3		Group 4		Group 5		Group 6	
Stimulus	A	B	A	B	A	B	A	B	A	B	A	B
**Y (cd/m^2^)**	4.44	4.50	4.91	4.99	9.09	9.09	3.20	3.19	3.06	3.04	8.12	8.16
**x**	0.362	0.362	0.280	0.283	0.364	0.366	0.286	0.285	0.260	0.258	0.319	0.321
**y**	0.284	0.286	0.336	0.336	0.381	0.384	0.260	0.259	0.264	0.264	0.393	0.394
**ΔL_ab_**	1.05		1.11		1.28		0.537		0.373		0.797	
**L-cone (%)**	23.9	24.2	24.3	24.8	47.0	47.1	16.3	16.2	15.2	15.1	40.7	40.9
**M-cone (%)**	21.5	21.9	29.3	29.7	49.0	49.0	17.9	17.8	18.2	18.1	47.1	47.3
**S-cone (%)**	45.2	45.1	45.8	46.2	49.7	48.3	45.6	45.9	45.1	45.0	48.6	48.0
**rod (%)**	29.8	29.9	38.8	39.0	54.1	53.6	28.6	28.4	28.8	29.0	54.0	53.6
**ipRGC (%)**	34.4	34.4	41.9	42.0	54.4	53.6	33.8	33.6	33.8	34.0	54.4	53.7

**Table A2:** x, y, cone, rod, and ipRGC stimulation amounts of theoretical pentamic-metamer stimuli. The rod and ipRGC stimulation amounts are defined as 100 for white light, the spectral distribution of which is 100 in the wavelength range.

	Group 1	Group 2	Group 3	Group 4	Group 5	Group 6
**x**	0.362	0.282	0.366	0.288	0.259	0.320
**y**	0.286	0.338	0.385	0.263	0.264	0.394
**L-cone (%)**	36.5	37.5	56.5	24.9	22.8	56.6
**M-cone (%)**	31.1	42.7	55.6	25.7	25.8	61.9
**S-cone (%)**	43.0	43.9	36.4	42.8	42.9	42.0
**rod (%)**	36.4	46.2	41.7	36.2	36.7	48.3
**ipRGC (%)**	40.3	51.1	46.2	40.1	40.7	53.5

Figure A2 shows the experimental flow. The experimental environment was the same as in the color discrimination experiment for the metameric ipRGC stimuli. The experiment was conducted on five participants in a color discrimination experiment of metameric ipRGC stimuli. Participants first observed a white stimulus with all spectral components of 100 for 10 s. Next, the test stimulus (stimulus A) was presented for 5 s. Following a chime sound, the stimulus was switched to the pentamic-metamer stimulus (stimulus B) with a 50% probability. Participants evaluated whether they perceived a color difference in the stimulus before and after the chime sound. If a participant could color discriminate pentamic-metamer stimuli, then the participant was capable of color discrimination even though the colors were the same for standard observers, and individual differences in visual characteristics may affect color discrimination. The test count was ten trials per stimulus pair. This meant that five out of ten trials were switched to the pentamic-metamer stimulus.

Figure A3 presents the results of the experiment. Figure A3a shows the percentage of perceived differences in the appearance of colors for each participant. As indicated by Figure A3a, two of the five participants (Participant 1 and 5) perceived a difference in appearance in the pentamic-metamer stimuli. Therefore, it is expected that these two participants were affected by individual differences in visual characteristics in the results of the experiment with metameric ipRGC stimuli. Additionally, Figure A3b shows the percentage of perceived differences in the appearance of colors for each Group (only Participants 2, 3 and 4). As shown in Figure A3b, Group 4’s experiment results were expected to be affected by individual differences in visual characteristics. On the other hand, Group 5’s stimuli were color discriminated in the experiment with metameric ipRGC stimuli, but not in the experiment with pentamic-metamer stimuli. Therefore, the results suggest that ipRGCs affect the color discrimination of blue.
